# Correlation of Anemia Due to Poor Iron Status With Obesity at King Fahad Medical City, Riyadh, Saudi Arabia

**DOI:** 10.7759/cureus.52424

**Published:** 2024-01-17

**Authors:** Ali A Alshehri, Odai M Albahli, Abdulrahman M Alturki, Turki A Alwasaidi, Nasreen F Alfaris

**Affiliations:** 1 Endocrinology and Metabolism, Endocrine and Metabolism Center, King Fahad Medical City, Riyadh, SAU; 2 Family Medicine, Saudi Board of Family Medicine, Kingdom of Saudi Arabia Ministry of Health, Riyadh, SAU; 3 Internal Medicine, King Fahad Medical City, Riyadh, SAU; 4 Internal Medicine, Prince Mohammed Bin Abdulaziz Hospital, Madinah, SAU; 5 Obesity, Endocrine, and Metabolism, King Fahad Medical City, Riyadh, SAU

**Keywords:** malnutrition, hemoglobin, iron deficiency anemia, body mass index, obesity

## Abstract

Background

Saudi Arabia has a high prevalence of chronic diseases such as obesity. Moreover, iron deficiency anemia (IDA) in developing countries is the most prevalent type of anemia. This study aims to assess the correlation between anemia related to poor iron status and obesity.

Methods

A cross-sectional observational study was conducted at the obesity center in King Fahad Medical City, Saudi Arabia, from April to September 2020. Two hundred and forty participants were needed to be included in the study. The data was gathered by utilizing a designed data collection form. Socio-demographic data, weight and height, questions related to the history of anemia, and gynecological data (for females) were collected. The data was analyzed using SPSS (Statistical Package for Social Science) version 28.0. Descriptive statistics were used to present numerical and categorical data and a Chi-square test was conducted to assess the correlation between categorical variables. Informed written consent was obtained from all participants and ethical approval was obtained from the Ethical Board Committee in King Fahad Medical City.

Results

The study included 240 participants. Two-thirds of the study population are females (64.6%), 66.7% are married, and 65.8% have obesity. Almost one-half of the study population (46%, N=128) was diagnosed with IDA with malnourishment being the most common reason for IDA (88.2%). The results indicated a correlation between obesity and the prevalence of IDA. The prevalence of IDA among participants with obesity (60.4%) was significantly higher compared to non-obese participants (39.5%), p=0.002. The study found that females and underweight individuals have a higher prevalence of IDA (p<0.001).

Conclusion

Results of the present study suggest that obesity could be associated with a risk of IDA. In addition, Saudi women could be more prone to IDA than men. Further prospective controlled studies among diverse populations in Saudi Arabia including laboratory assessment of inflammatory markers and iron status are required to better understand the correlation between obesity and IDA.

## Introduction

Anemia is a medical condition characterized by a shortage of red blood cells, which causes a hindrance in the transportation of oxygen throughout the body. Anemia is often caused by iron deficiency. It can occur because of inadequate intake of iron, poor absorption of iron in the body, or blood loss [1].

Various factors (modifiable and non-modifiable) can affect an iron balance in the body. These factors are age, education, income, marital status, gender, impact on various aspects of life, ethnicity, and the quality and amount of food an individual consumes. Iron balance is also affected by physical and mental health, medications, and underlying medical conditions, in addition to genetic factors [2].

Iron deficiency anemia (IDA) is a serious health condition caused by an untreated lack of iron. Tiredness, reduced productivity, and poor maternal health are some of the consequences associated with this issue, particularly among pregnant women, inadequate cognitive functions, and even delayed motor and mental development in children. It is still unclear whether short-term treatment can alter these outcomes [3-7].

The World Health Organization (WHO) has reported that 24.8% of the world population (1.6 billion people) are affected by anemia. Iron deficiency is twice as prevalent as IDA worldwide [8]. In Saudi Arabia, the prevalence of IDA was 30%-56% [9].

Previous studies revealed that obesity could be associated with iron deficiency or iron-related disorders. This is caused by various factors, such as low iron intake, poor iron bioavailability, and insufficient release and uptake of iron stores which are affected by the overexpression of hepcidin [10,11].

Several studies that assess the association between iron deficiency and obesity focused mainly on specific hematological or biochemical markers to evaluate iron status. These factors included serum iron level, hemoglobin concentration, hematocrit, and ferritin. In contrast, more studies are needed to assess inflammatory markers and serum hepcidin due to their vital role in iron homeostasis [12].

The latest global projections from WHO show that approximately 1.6 billion adults were affected by pre-obesity, while 400 million adults had obesity in 2005, with a relatively high prevalence of obesity and pre-obesity (74% to 86%) among women living in the Eastern Mediterranean Region [13,14].

It is estimated that 40%-46% of adolescents in the Arabian Gulf states are overweight or obese. These are among the highest prevalence in the world [15]. Saudi Arabia has a high prevalence of chronic diseases, including obesity. Memish et al. evaluated 10735 adult participants from 13 regions of Saudi Arabia. It has been found that 28.7% of individuals had obesity with a body mass index (BMI) of ≥30 kg/m^2^ [16].

No previous studies in Saudi Arabia correlated IDA with obesity among the adult population, including males. The prior studies focused on adult females, adolescents, and children [17-19]. Therefore, this study aimed to investigate the relationship between IDA and obesity among adults in Saudi Arabia, a region with a high prevalence of obesity. The results of the current study can help primary care physicians and family physicians predict the risk of IDA among patients with obesity. Early screening and detection of IDA can significantly improve the health status and quality of life of this patient population.

## Materials and methods

Study design

A cross-sectional observational study was carried out from 15 April to 17 September 2020. We followed a quota sampling technique. All potential study subjects were screened for participation in the study.

Study population

Adult Saudi residents of both genders seen at the obesity center in King Fahad Medical City, Saudi Arabia, during the data collection period were included in the present study. Participants aged less than 18 years and those with hereditary blood diseases such as hemoglobinopathy, thalassemia, enzyme deficiencies, membrane defects, and bone marrow failure were excluded from the study. In addition, patients with chronic IDA related to chronic bleeding (gynecological or GI related) or post-bariatric surgery and patients with anemia resulting from chronic diseases, e.g., diabetes mellitus, heart failure, connective tissue disease, chronic kidney disease, inflammatory bowel disease, or any active chronic infectious disease, were also excluded from the study.

Data collection

The data was gathered by utilizing a designed data collection form and included (1) socio-demographic data such as age, gender, weight, height, marital status, and nationality. Data was collected through anthropometric measures. Participants’ heights and weights were measured, while standing straight on a calibrated scale, barefoot, and wearing minimal clothing. Furthermore, BMI was calculated by dividing weight in kilograms by the square of height in meters (kg/m²). In the current study, a BMI of less than 18.5 kg/m^2^ is considered underweight, while a BMI of 18.5 to 24.9 kg/m^2^ is considered normal weight. A BMI from 25 to 29.9 kg/m^2^ is classified as overweight, whereas a BMI of 30 kg/m^2^ or more is considered as obesity. (2) Questions related to the history of anemia, such as diagnosis with IDA and its reason; treatment with an iron supplement or receiving blood transfusion; other causes of anemia such as autoimmune blood disorder, bone marrow disease, celiac disease, anemia with B12 deficiency. (3) Gynecological factors among female participants: being pregnant, type of delivery, suffering from heavy menstruation, and taking contraceptive pills.

Sample size

In the study, 240 participants were required to be included. The sample size was determined by applying the following formula: Z 1-α/2 2 SD2/ d2. In this formula, Z1-α/2 represents the standard normal variate for a 5% type 1 error, SD is the standard deviation, and d is the absolute precision that has been taken as 0.05.

Statistical analysis

The data was gathered and evaluated using SPSS (Statistical Package for Social Science) version 28.0 (IBM Corp., Armonk, NY). The numerical data was presented using mean and standard deviation (SD), while the categorical data was shown as frequency and percentage. To determine the correlation between categorical variables, a chi-square test was conducted. The statistical significance was established by considering p-values below 0.05.

Ethical consideration

Informed written consent was obtained from all participants. The study objectives and methodology were explained, patient privacy was ensured, and data confidentiality was maintained. The Ethical Board Committee in King Fahad Medical City granted ethical approval in April 2020 with an approval IRB number: 20-061. Participants confidentiality was assured, and no personal data was collected. The collected data was saved in a secured cabinet and would be kept for five years in King Fahad Medical City.

## Results

Study population

In the current study, 240 participants completed the data collection form. Participants’ mean age (SD) was 35.3 (9.9) years. Almost two-thirds of the participants (64.6%) were females, 66.7% were married, and 65.8% had obesity (BMI>30 kg/m2). The majority of respondents were Saudi (91.2%). All data are shown in Table 1.

**Table 1:** Socio-demographics of study participants The data was represented as mean ± standard deviation for numerical data and as number (percentage) for categorical data.

Characteristics	Total (N=240), Count (%)
Age (mean±SD )	35.34±9.915
Gender	
Male	85 (35.4)
Female	155 (64.6)
Marital status	
Single	61 (26.8)
Married	152 (66.7)
Divorced	10 (4.4)
Widowed	5 (2.2)
BMI	
Underweight	17 (7.1)
Healthy weight	32 (13.3)
Pre-obesity	33 (13.8)
Obesity	158 (65.8)
Nationality	
Saudi	201 (91.2)
Other nationality	18 (8.8)

History of anemia and comorbidities among participants

Of 240 participants included in this study, 46% (N=128) had IDA. The most common reason for IDA was malnourishment (88.2%, N=90). Most respondents were treated with iron supplementation (60.6%, N=145). Additionally, individuals diagnosed with hereditary anemia and autoimmune blood/bone marrow disease represented only 0.8% (N=2) of the population. Details are fully illustrated in Table 2.

**Table 2:** History of anemia and comorbidities among participants (N=240) The data was represented as a number (percentage).

1) Diagnosed with IDA		Total, N (%)
Yes		128 (46)
No		112 (54)
Reason for IDA		
Excessive bleeding during menstruation		14 (13.7)
Malnourishment		90 (88.2)
Unknown		5 (4.9)
Treated with iron supplementation		
Yes		94 (39.4)
No		145 (60.6)
Type of iron supplementation		
IV for one month		1 (0.4)
IV for two months		1 (0.4)
Pills for days		1 (0.4)
Pills for one week		19 (7.9)
Pills for one month		54 (22.5)
Pills for two months		11 (4.6)
Pills for months		1 (0.4)
Blood transfusion		5 (2)
2) Diagnosed with hereditary anemia		2 (0.8)
3) Diagnosed with hereditary or acquired bleeding disorder		1 (0.4)
4) Diagnosed with anemia due to B12 deficiency		1 (0.4)
5) Diagnosed with celiac disease		1 (0.4)
6) Diagnosed with hereditary/acquired autoimmune blood or bone marrow disease		2 (0.8)
7) Treated with blood thinners		1 (0.4)

Gynecological factors among female participants

Regarding gynecological considerations among female individuals, almost 58% (N=90) had no previous birth, whereas 25% (N=39) had less than three children. Additionally, normal vaginal delivery was more than cesarean section. Also, females with heavy bleeding during menstruation and during or after birth were 1.9% (N=3) and 0.7% (N=1), respectively. More details are provided in Table 3.

**Table 3:** Gynecological factors among female participants The data was represented as a number (percentage)

	Count (%), Female, N=155
Current pregnancy	0 (0)
Previous births	
No	90 (58.1)
Less than 3	39 (25.1)
More than 3	15 (9.7)
Type of previous delivery	
Normal vaginal delivery	2 (1.3)
Cesarean section	1 (0.7)
Heavy bleeding during menstruation	3 (1.9)
Heavy bleeding during or after birth	1 (0.7)

Association between BMI and IDA

The results showed that patients with obesity had a significantly higher incidence of IDA than patients who did not have obesity (p=0.002) (Table 4). By sub-analyzing respondents with BMI, a significantly larger proportion of patients with IDA was found among underweight and individuals with obesity (p<0.001). All details are illustrated in Table 5.

**Table 4:** Prevalence of IDA among participants with obesity and participants without obesity Non-obese participants <30 kg/m^2^, obese participants ≥30 kg/m^2^ The data was represented as a number (percentage), and the association was represented as a p-value, where the p-value was considered statistically significant when it was less than 0.05.

BMI	Diagnosed with iron deficiency anemia		P-value
	Yes (N=128)	No (N=112)	
Non-obese	32 (39.5)	49 (60.5)	0.002
Obese	96 (60.4)	63 (39.6)	

**Table 5:** Association between obesity and IDA The data was represented as a number (percentage), and the association was represented as a p-value, where the p-value was considered statistically significant when it was less than 0.05.

BMI	Diagnosed with iron deficiency anemia		P-value
	Yes, N (%) (N=128)	No, n (%) (N=112)	
Underweight	15 (93.7)	1 (6.3)	<0.001
Healthy weight	7 (21.9)	25 (78.1)	
Overweight	10 (50)	10 (50)	
Obesity	96 (60.4)	63 (39.6)	

Association between IDA and gender

By assessing the relationship between gender and IDA, the prevalence of IDA among females (63.9%, N=99) was significantly higher (p<0.001) compared to males (34.1%, N=29), as shown in Table 6.

**Table 6:** Distribution of IDA among different genders The data was represented as a number (percentage), and the association was represented as a p-value, where the p-value was considered statistically significant when it was less than 0.05.

Gender	Diagnosed with iron deficiency anemia		P-value
	Yes (N=128)	No (N=112)	
Female	99 (63.9%)	56 (36.1%)	<0.001
Male	29 (34.1%)	56 (65.9%)	

Upon investigating the association between obesity and IDA among the different genders, female participants with BMI ≥30 mg/kg^2^ were found to have more IDA than females without obesity. However, the association between BMI and IDA was not statistically significant (p=0.069). It was observed that male participants with BMI <30 kg/m^2^ had a lower prevalence of IDA (75%, N=30). Also, the relationship was not significant (p=0.095), as shown in Table 7.

**Table 7:** Correlation between IDA and obesity classified by gender Non-obese participants <30 kg/m^2^, obese participants ≥30 kg/m^2^ The data was represented as a number (percentage), and the association was represented as a p-value, where the p-value was considered statistically significant when it was less than 0.05.

Gender	Obesity	Diagnosed with iron deficiency anemia		P-value
		Yes (N=128)	No (N=112)	
Female	No obesity	22 (52.4%)	20 (47.6%)	0.069
	With obesity	77 (68.1%)	36 (31.9%)	
Male	No obesity	10 (25%)	30 (75%)	0.095
	With obesity	19 (42.2%)	26 (57.8)	

## Discussion

Obesity and IDA remain significant public health concerns globally. The correlation between IDA and obesity using BMI has been revealed in many countries [20]. In Saudi Arabia, obesity is a chronic disease with a high prevalence that subsequently may increase the incidence of IDA and its burdens [16]. In addition, several observational studies have examined the relationship between increasing BMI and IDA among women and children [17-19]. No prior studies assessing the association between IDA and obesity in Saudi Arabia among the adult population, including both genders, were established. Accordingly, the present study aimed to address the relationship between IDA and obesity among Saudi adults.

In the current study, an inverse relationship between obesity and IDA was suggested. The results obtained from the study were in line with those of a cross-sectional study in Washington, USA. The latter study examined the relationships between obesity and serum iron in adults with obesity and adults without obesity. The observations show an association between obesity and iron deficiency [21]. Another cross-sectional study in Minneapolis used baseline data from a randomized clinical trial on individuals living with obesity and subjects without the disease of obesity. Serum iron levels were significantly reduced in individuals with obesity in comparison to those without obesity [22].

Furthermore, our findings demonstrated that underweight individuals significantly correlated with IDA disease. This could be justified due to lower iron intake in underweight individuals.

As seen in our results, the relationship between gender and IDA was illustrated. It was found that the prevalence of females who have IDA was statistically higher than males who were diagnosed with IDA. Prior studies revealed that one of the leading causes of IDA is sex-related, with heavy menstrual loss being the most common reason for IDA in women of reproductive age [23,24].

Multiple studies were conducted solely on females as they have a higher prevalence of IDA than males. A previous study in Bangladesh in 2020 revealed high rates of pre-obesity and obesity among urban women who suffer from IDA [25]. In Egypt, a cross-sectional study aimed to investigate the association of IDA with BMI among adolescent females. The study results suggested that females with pre-obesity had the lowest percentage of anemic participants. However, this finding contradicts the results of our study [26].

Moreover, our findings did not reveal a significant difference between IDA and obesity (≥30 kg/m2) among females and males. Our results were in accordance with a population-based study in Nigeria in 2018. The trial aimed to explore the correlation between serum phosphate and iron levels with BMI. Their results showed that the mean serum iron levels of pre-obesity in subjects with severe obesity were statistically lower than in participants with healthy weight in both males and females. The differences could be due to diverse methodologies. Our study included underweight subjects (BMI <18.5 kg/m^2^), unlike the Nigerian study. Based on our previous findings, a higher prevalence of IDA in underweight participants was seen among our population [27].

One of the limitations of the current study is being cross-sectional in design, making it difficult to confirm the causality between IDA and obesity. In addition, there is a lack of necessary laboratory tests to evaluate the iron status of the participants.

## Conclusions

IDA is a serious global public health problem worldwide. This cross-sectional study demonstrated that obesity could be associated with a risk of IDA in the Saudi Arabian community. A possible relationship is seen between being underweight and the probability of having IDA. In addition, Saudi women could be more prone to IDA than men. However, among females and males, there is no association between IDA and obesity. Further studies, including laboratory assessment of inflammatory markers and iron status, are recommended to provide more robust evidence about the correlation between obesity and IDA and to validate the observed associations. In addition, further research across diverse populations in Saudi Arabia is required to enhance the external validity of the findings.

## References

[1] Powers JM, Buchanan GR (2014). Diagnosis and management of iron deficiency anemia. Hematol Oncol Clin North Am.

[2] Al-Quaiz JM (2001). Iron deficiency anemia. A study of risk factors. Saudi Med J.

[3] Percy L, Mansour D, Fraser I (2017). Iron deficiency and iron deficiency anaemia in women. Best Pract Res Clin Obstet Gynaecol.

[4] Halib H, Muda WM, Dam PC, Mohamed HJ (2017). Prevalence of iron deficiency and its associated risk factors among primary school children in Kelantan. Int J Fundam Appl Sci.

[5] Bah F, Harith S, Farisni TN (2020). Food knowledge and practices related to anemic conditions among pregnant women in Kuala Terengganu, Malaysia. J-Kesmas: Jurnal Fakultas Kesehatan Masyarakat (The Indones. J. Public Health).

[6] Zani H, Shahril MR, Rahman WN, Mukhali HB, Ismail R, Yusop YM (2020). Anaemia-related knowledge amongst pregnant women in Kuala Terengganu, Malaysia. Asian J Med Biomed.

[7] Wang B, Zhan S, Gong T, Lee L (2013). Iron therapy for improving psychomotor development and cognitive function in children under the age of three with iron deficiency anaemia. Cochrane Database Syst Rev.

[8] McLean E, Cogswell M, Egli I, Wojdyla D, de Benoist B (2009). Worldwide prevalence of anaemia, who vitamin and mineral nutrition information System, 1993-2005. Public Health Nutr.

[9] Verster A, Vander Pols JC (1995). Anaemia in the Eastern Mediterranean region. East Mediterr Health J.

[10] Weiss G, Goodnough LT (2005). Anemia of chronic disease. N Engl J Med.

[11] Brotanek JM, Gosz J, Weitzman M, Flores G (2007). Iron deficiency in early childhood in the United States: risk factors and racial/ethnic disparities. Pediatrics.

[12] Aderibigbe OR, Pisa PT, Vorster HH, Kruger SH (2014). The relationship between iron status and adiposity in women from developing countries: a review. Crit Rev Food Sci Nutr.

[13] (2010). World Health Organization. http://apps.who.int/bmi/index.jsp.

[14] WHO WHO (2019). Obesity and overweight. World Health Organization.

[15] Ng SW, Zaghloul S, Ali HI, Harrison G, Popkin BM (2011). The prevalence and trends of overweight, obesity and nutrition-related non-communicable diseases in the Arabian Gulf States. Obes Rev.

[16] Memish ZA, El Bcheraoui C, Tuffaha M (2014). Obesity and associated factors—Kingdom of Saudi Arabia, 2013. Prev Chronic Dis.

[17] Fatin AS, Mamdooh G, Safaa Q, Nadiah B, Adel A (2011). Prevalence of iron deficiency and iron deficiency anemia among females at university stage. J Med Lab Diagn.

[18] Alquaiz AJ, Khoja TA, Alsharif A (2015). Prevalence and correlates of anaemia in adolescents in Riyadh city, Kingdom of Saudi Arabia. Public Health Nutr.

[19] Aloufi ME, Aljaed NM, Aloufi RA, Jafri SA, Elnashar MA (2018). Prevalence of Iron Deficiency anemia in Obese Children in Taif Area-Saudi Arabia. Egypt J Hosp Med.

[20] Eckhardt CL, Torheim LE, Monterrubio E, Barquera S, Ruel MT (2008). The overlap of overweight and anaemia among women in three countries undergoing the nutrition transition. Eur J Clin Nutr.

[21] Yanoff LB, Menzie CM, Denkinger B, Sebring NG, McHugh T, Remaley AT, Yanovski JA (2007). Inflammation and iron deficiency in the hypoferremia of obesity. Int J Obes (Lond).

[22] Menzie CM, Yanoff LB, Denkinger BI, McHugh T, Sebring NG, Calis KA, Yanovski JA (2008). Obesity-related hypoferremia is not explained by differences in reported intake of heme and nonheme iron or intake of dietary factors that can affect iron absorption. J Am Diet Assoc.

[23] Goddard AF, McIntyre AS, Scott BB (2000). Guidelines for the management of iron deficiency anaemia. Gut.

[24] Pasricha SR, Flecknoe-Brown SC, Allen KJ (2010). Diagnosis and management of iron deficiency anaemia: a clinical update. Med J Aust.

[25] Ali NB, Dibley MJ, Islam S (2021). Overweight and obesity among urban women with iron deficiency anaemia in Bangladesh. Matern Child Nutr.

[26] ELMoslemany AG, ELBbandrawy AM, Elhosary EA, Gabr AA (2019). Relation between body mass index and iron deficiency anemia in adolescent females. Curr Sci Int.

[27] Egwurugwu JN, Ekweogu CN, Nwamkpa P, Ohamaeme MC, Ugwuezumba PC, Ogunnaya FU (2018). Association between serum phosphate and iron concentrations with body mass index in a population of adults in Orlu, Imo State, Nigeria. Nigerian J Exp Clin Biosci.

